# Quorum Sensing Activity of a *Kluyvera* sp. Isolated from a Malaysian Waterfall

**DOI:** 10.3390/s140508305

**Published:** 2014-05-08

**Authors:** Nina Yusrina Muhamad Yunos, Wen-Si Tan, Nur Izzati Mohamad, Pui-Wan Tan, Tan-Guan-Sheng Adrian, Wai-Fong Yin, Kok-Gan Chan

**Affiliations:** Division of Genetics and Molecular Biology, Institute of Biological Sciences, Faculty of Science, University of Malaya, 50603 Kuala Lumpur, Malaysia; E-Mails: ninayusrina@hotmail.com (N.Y.M.Y.); tmarilyn36@gmail.com (W.-S.T.); zetty_mohamad@yahoo.com (N.I.M.); acelinetan38@yahoo.com (P.-W.T.); adrian_tan_1991@yahoo.com (T.-G.-S.A.); yinwaifong@yahoo.com (W.-F.Y.)

**Keywords:** cell-to-cell communication, mass spectrometry, triple quadrupole liquid chromatography mass spectrometry, *N*-(3-oxohexanoyl)homoserine lactone, *N*-3-oxo-octanoyl-L-homoserine lactone (3-oxo-C8 HSL), *Kluyvera* sp

## Abstract

In many species of bacteria, the quorum sensing mechanism is used as a unique communication system which allows them to regulate gene expression and behavior in accordance with their population density. *N*-Acylhomoserine lactones (AHLs) are known as diffusible autoinducer molecules involved in this communication network. This finding aimed to characterize the production of AHL of a bacterial strain ND04 isolated from a Malaysian waterfall. Strain ND04 was identified as *Kluyvera* sp. as confirmed by molecular analysis of its 16S ribosomal RNA gene sequence. *Kluyvera* sp. is closely related to the Enterobacteriaceae family. *Chromobacterium violaceum* CV026 was used as a biosensor to detect the production of AHL by strain ND04. High resolution triple quadrupole liquid chromatography-mass spectrometry analysis of strain ND04 showed our isolate produced two AHLs which are *N*-(3-oxohexanoyl)homoserine lactone (3-oxo-C6 HSL) and *N*-3-oxo-octanoyl-L-homoserine lactone (3-oxo-C8 HSL).

## 1. Introduction

Quorum sensing (QS) is a cell-to-cell communication mechanism used by many species of bacteria [1,2] in order to measure the density of their own population within their environment and to regulate their gene expression and behavior accordingly [3]. QS bacteria produce and release self-generated signal molecules called autoinducers that increase in concentration as a function of cell density [4–6]. According to Winzer and colleagues, QS was first proposed by Fuqua *et al.* which describe the following mechanism in detail [2]. The detection of a minimal threshold stimulatory concentration of an autoinducer leads to an alteration in gene expression [4].

In order to regulate a diverse array of physiological activities such as symbiosis, virulence, competence, conjugation, antibiotic production, motility, sporulation and biofilm formation, both Gram-positive and Gram-negative bacteria use QS communication circuits [4]. To date, there have been more studies on cell-to-cell communication between Gram-negative bacteria than Gram-positive ones. In general, the vast majority of Gram-negative bacteria QS systems that have been studied use *N*-acylhomoserine lactones (AHL) as autoinducers [5]. LuxI and LuxR regulatory proteins are involved in QS of Gram-negative bacteria [1]. The biosynthesis of specific signaling molecules, acyl-homoserine lactones (AHLs), also known as autoinducers, is regulated by the LuxI-like proteins. The concentration of autoinducers increases as the cell population density increases [7]. LuxR-like protein is the receptor which will bind cognately to the autoinducers when the signal reaches a critical threshold level [8]. Therefore, these LuxR-autoinducer complexes specifically regulate gene transcription [9].

QS bacteria have adapted to almost everywhere and the aquatic environment could be a reservoir for them. In this study, we have isolated from a Malaysian waterfall the strain ND04 that shows QS properties. It has been identified as *Kluyvera* sp. According to Farmer *et al.*, *Kluyvera* strains share the properties of most members of the Enterobacteriaceae family which are Gram-negative, rod-shaped, catalase positive, motile with peritrichous flagella and oxidase negative [10]. In 1988, Luttrell and co-workers reported the first case of soft tissue infection in a healthy woman caused by *Kluyvera* sp. However, compromised hosts are the most affected [11]. Hence, in order to develop therapeutic agents to treat these bacteria, it is important to have better understanding of *Kluyvera* sp. In this paper, we aimed to identify the AHL(s) produced by *Kluyvera* sp. strain ND04 isolated from a Malaysian waterfall.

## 2. Experimental Section

### 2.1. Water Sample Collection

Water samples were collected from the Sungai Ampang waterfalls, Ulu Klang, Selangor, Malaysia in October 2013. The samples were collected at a depth of 12–17 cm. Samples were collected in sterilized plastic tubes. At the collection spot, physical temperatures and pH of the samples were recorded. An icebox were used to store the water samples and brought to the University of Malaya for further analysis. The samples were stored in a refrigerator at 4 °C till further processing [12].

### 2.2. Isolation of Bacterial Strains

A 10 μL tenfold serial dilution (10^−1^, 10^−2^, 10^−3^, 10^−4^) of the overnight culture was spread on Reasoner's 2A (R2A) agar [13]. Then the plates were incubated at 28 °C for 24 h. After 24 h, visibly distinguishable bacterial colonies were identified and each colony was transferred onto Trypticase Soy Agar (TSA) by streaking using a sterile inoculating loop. The plates were then incubated again at 28 °C for 24 h. At this point, the original observations about the size, shape, color as well as other visual properties of each isolate were recorded.

### 2.3. Strain Identification using 16S rRNA

**Figure 1.**
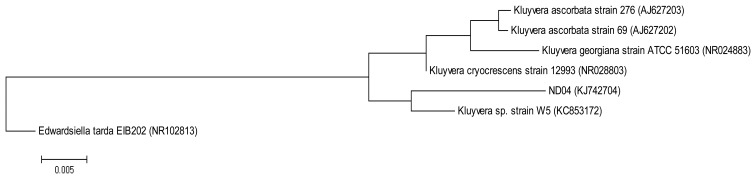
The evolutionary history was inferred by using the Maximum Likelihood method based on the Tamura-Nei model. The tree with the highest log likelihood (−2,463.0212) is shown. Initial tree(s) for the heuristic search were obtained automatically by applying Neighbor-Join and BioNJ algorithms to a matrix of pairwise distances estimated using the Maximum Composite Likelihood (MCL) approach, and then selecting the topology with superior log likelihood value. The tree is drawn to scale, with branch lengths measured in the number of substitutions per site. The analysis involved seven nucleotide sequences. Codon positions included were 1st + 2nd + 3rd + Noncoding. All positions containing gaps and missing data were eliminated. There were a total of 1,295 positions in the final dataset. Evolutionary analyses were conducted in MEGA 6. *Edwardsiella tarda* EIB202 was used as an outgroup. Numbers in parentheses are GenBank accession numbers.

The QIAamp^®^ DNA Mini Kit (Qiagen, Germantown, MD, USA) was used to extract and purify bacterial genomic DNA and the resulting DNA was used as a template for PCR. The forward primer 27F (5′-AGAGTTTGATCMTGGCTCAG-3′) and the reverse primer 1525R (5′-AAGGAGGTGWTCCARCC-3′) were used to amplify the 16S rRNA gene [14]. The following PCR conditions were used: initial denaturation at 94 °C for 5 min, followed by 30 cycles at 94 °C for 30 s, annealing at 63 °C for 30 s and extension at 72 °C for 1 min 30 s, and a final extension at 72 °C for 5 min [15]. The 16S rRNA gene sequence of strain ND04 was aligned with sequences of closely related type strains retrieved from the GenBank database. A phylogenetic tree (Figure 1) was reconstructed using the maximum likelihood algorithm. Evolutionary analyses were conducted in Molecular Evolutionary Genetic Analysis (MEGA) version 6 [16,17].

### 2.4. AHL Detection

*Chromobacterium violaceum* CV026 was used as an AHL biosensor which detect the presence of exogenous short chain AHLs ranging from four to eight carbons [18]. A purple pigmentation will be induced in *C. violaceum* CV026 if any AHL molecules are present [14,18]. The cross streak method was used to screen the bacteria isolates with *C. violaceum* CV026 on Luria Bertani (LB) Agar. *Erwinia carotovora* GS101 were used as positive control while *E. carotovora* PNP22 was the negative control [19,20].

### 2.5. AHL Extraction

Bacterial colonies that showed positive results for the detection of AHL were grown overnight in LB broth buffered to pH 6.5 with 3-(*N*-morpholino)propanesulfonic acid (MOPS, 50 mM, pH 6.5) at 28 °C with shaking (220 rpm). An equal volume of acidified (0.1% v/v acetate acid) ethyl acetate was used to extract the spent supernatant twice [21]. The AHL extracts were then dried in a fume hood and stored at −20 °C for further analysis using liquid chromatography-mass spectrometry (LC/MS).

### 2.6. AHL Identification by Triple Quadrupole Liquid Chromatography Mass Spectrometry (LC/MS)

Extracted AHLs were reconstituted in acetonitrile followed by LC/MS analysis using an Agilent 1290 Infinity LC system (Agilent Technologies, Santa Clara, CA, USA) equipped with an Agilent ZORBAX Rapid Resolution High Definition SB-C18 Threaded Column (2.1 mm × 50 mm, with packed particle size of 1.8 μm) [20]. Respectively, 0.5 mL/min was set as the flow rate at 37 °C and the injection volume was 2 μL. Mobile phases A and B refer to water and acetonitrile, respectively, where both containing 0.1% v/v formic acid). The gradient profile was set at A:B 80:20 at 0 min, 50:50 at 7 min, 20:80 at 12 min, and 80:20 at 14 min. Subsequent MS detection of separated compounds was performed on the Agilent 6490 Triple Quadrupole LC/MS system. Precursor ion-scanning analysis were performed in positive ion mode with Q3 set to monitor for *m/z* 102 and Q1 set to scan a mass range of *m/z* 80 to 400. Molecular mass of *m/z* 102 is characteristics of the lactone ring moiety thus indicating presence of AHLs. The MS parameters used are: probe capillary voltage set at 3 kV, sheath gas at 11 mL/h, nebulizer pressure of 20 pounds per square inch (p.s.i.) and desolvation temperature of 200 °C [22]. The Agilent Mass Hunter software was used for the MS data analysis to confirm the presence of AHLs [22]. Analysis was based on the retention index and the comparison of the EI mass spectra with AHL standards.

## 3. Results and Discussion

### 3.1. Identification of Bacterial Isolates

On sampling day, the temperature and the pH value of the water collected were 25.5 °C and pH 8, respectively. After several successive streaking, pure colonies were obtained. Strain ND04 was isolated from one of the single colonies and identified using 16S rRNA gene sequencing. The sequence was subsequently deposited into GenBank with the accession number KJ742704. According to a web-based search and phylogenetic analysis of the 16S rRNA gene sequence strain ND04 showed 99% similarity to *Kluyvera* sp. Strain W5 (Figure 1). The range of identity similarity between reference strains and ND04 is between 96%–99%.

### 3.2. Production of AHLs

**Figure 2.**
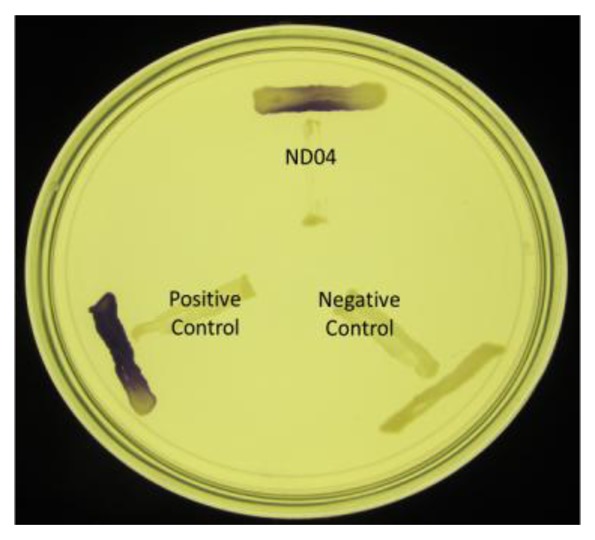
Cross streak of strain ND04 with *C. violaceum* CV026. *E. carotovora* GS101 (positive control) and *E. carotovora* PNP22 (negative control) that produces AHL and depleted AHL production, respectively, were included as controls.

**Figure 3.**
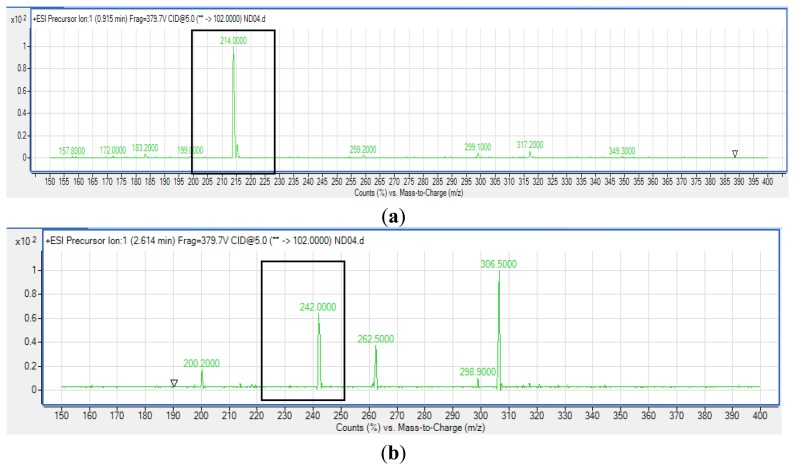
Identification of 3-oxo-C6-HSL (*m/z* 214.0000) (**a**) (boxed) and 3-oxo-C8-HSL (*m/z* 242.0000) (**b**) (boxed) from AHL extract of the spent culture supernatant of strain ND04 by Triple Quadrupole LC/MS.

Strain ND04 was screened for the production of AHLs by using *C. violaceum* CV026. Strain ND04 showed a positive result by triggering *C. violaceum* CV026 violacein production suggesting the production of short chain AHLs (Figure 2). Subsequently, high resolution triple quadrupole LC/MS system was used in order to identify the AHLs. The presence of two AHLs was confirmed by the mass spectrometry analysis, namely 3-oxo-C6-HSL (*m/z* 214.0000) and 3-oxo-C8-HSL (*m/z* 242.0000) (Figure 3).

The detection of AHLs in *Kluyvera* sp. strain ND04, remarkably 3-oxo-C6-HSL and 3-oxo-C8-HSL, is the first report on this finding. This study of the QS mechanism of *Kluyvera* sp. strain ND04 has significance whereby understanding it may allow the control of disease by disrupting their communication. Hence, more intense research onto the mechanism of QS in *Kluyvera* sp. should be performed in the near future for this purpose.

## 4. Conclusions/Outlook

A bacterial strain ND04 that has been isolated from a Malaysian waterfall belongs to *Kluyvera* sp. as confirmed by 16S rDNA nucleotide analysis. It shows QS activity by producing 3-oxo-C6-HSL and 3-oxo-C8-HSL, as confirmed by LC/MS analysis.

## References

[1] Bharati B.K., Chatterji D. (2013). Quorum sensing and pathogenesis: Role of small signalling molecules in bacterial persistence. Curr. Sci..

[2] Winzer K., Hardie K.R., Williams P. (2002). Bacterial cell-to-cell communication: Sorry, can't talk now—Gone to lunch. Curr. Opin. Microbiol..

[3] Popham D.L., Stevens A.M. (2005). Bacterial quorum sensing and bioluminescence. 2005 Proc. Assoc. Biol. Lab. Educ. (ABLE).

[4] Miller M.B., Bassler B.L. (2001). Quorum sensing in bacteria. Ann. Rev. Microbiol..

[5] de Kievit T.R., Iglewski B.H. (2000). Bacterial quorum sensing in pathogenic relationship. Infect. Immun..

[6] Antunes L.C.M., Ferreira R.B.R., Buckner M.M.C., Finlay B.B. (2010). Quorum sensing in bacterial virulence. Microbiology.

[7] Waters C.M., Bassler B.L. (2005). Quorum sensing: Cell-to-cell communication in bacteria. Annu. Rev. Cell Dev. Biol..

[8] Zheng H., Zhong Z., Lai X., Chen W.X., Li S., Zhu J. (2006). A luxr/luxi-type quorum-sensing system in a plant bacterium, *Mesorhizobium tianshanense*, controls symbiotic nodulation. J. Bacteriol..

[9] Galloway W.R.J.D., Hodgkinson J.T., Bowden S.D., Welch M., Spring D.R. (2011). Quorum sensing in gram-negative bacteria: Small-molecule modulation of AHL and AI-2 quorum sensing pathways. Chem. Rev..

[10] Farmer J.J., Fanning G.R., Huntley-Carter G.P., Holmes B., Hickman F.W., Richard C., Brenner D.J. (1981). *Kluyvera*, a new (redefined) genus in the family *Enterobacteriaceae*: Identification of *Kluyvera ascorbata* sp. nov. and *Kluyvera cryocrescens* sp. nov. in clinical specimens. J. Clin. Microbiol..

[11] Luttrell R.E., Rannick G.A., Hernandez J.L.S., Verghese A. (1988). *Kluyvera* species soft tissue infection: Case report and review. J. Clin. Microbiol..

[12] Parida S., Jena R.C., Samal K.C., Chand P.K. (2012). Isolation and identification of pathogenic bacteria from brackish waters of Chilika Lagoon, Odisha, India for pharmaceutical use. Mal. J. Microbiol..

[13] Stetzenbach L.D., Kelley L.M., Sinclair N.A. (1986). Isolation, identification and growth of well-water bacteria. Natl. Ground Water Assoc..

[14] Chen J.W., Koh C.L., Sam C.K., Yin W.F., Chan K.G. (2013). Short chain *N*-acyl Homoserine Lactone Production by Soil Isolate *Burkholderia* sp. Strain A9. Sensors.

[15] Wong C.S., Koh C.L., Sam C.K., Chen J.W., Chong Y.M., Yin W.F., Chan K.G. (2013). Degradation of bacterial quorum sensing signaling molecules by the microscopic yeast *Trichosporon Loubieri* isolated from tropical wetland waters. Sensors.

[16] Tamura K., Nei M. (1993). Estimation of the number of nucleotide substitutions in the control region of mitochondrial DNA in humans and chimpanzees. Mol. Bio. Evol..

[17] Tamura K., Stecher G., Peterson D., Filipski A., Kumar S. (2013). MEGA6: Molecular Evolutionary Genetics Analysis Version 6.0. Mol. Biol. Evol..

[18] McClean K.H., Winson M.K., Fish L., Taylor A., Chhabra S.R., Camara M., Daykin M., Lamb J.H., Swift S., Bycroft B.W. (1997). Quorum sensing and *Chromobacterium violaceum*: Exploitation of violacein production and inhibition for the detection of *N*-acylhomoserine lactones. Microbiology.

[19] Lau Y.Y., Sulaiman J., Chen J.W., Yin W.F., Chan K.G. (2013). Quorum sensing activity of *Enterobacter asburiae* isolated from lettuce leaves. Sensors.

[20] McGowan S., Sebaihia M., Jones S., Yu B., Bainton N., Chan P.F., Bycroft B., Stewart G.S.A.B., Williams P., Salmond G.P.C. (1995). Carbapenem antibiotic production in *Erwinia carotovora* is regulated by CarR, a homologue of the LuxR transcriptional activator. Microbiology.

[21] Zhu H., Thuruthyil S.J., Willcox M.D.P. (2002). Determination of quorum-sensing signal molecules and virulence factors of *Pseudomonas aeruginosa* isolates from contact lens-induced microbial keratitis. J. Med. Microbiol.

[22] Yin W.F., Purmal K., Chin S., Chan X.Y., Koh K.L., Sam C.K., Chan K.G. (2012). *N*-acyl homoserine lactone production by *Klebsiella pneumoniae* isolated from human tongue surface. Sensors.

